# Supplementary material for: Stress-induced plasticity of a CRH/GABA projection disrupts reward behaviors in mice
Source: Nat Commun. 2023 Feb 25;14:1088. doi: 10.1038/s41467-023-36780-x (PMC9968307; doi:10.1038/s41467-023-36780-x)
Supplement: Supplementary file 1 — Supplementary Information [file 41467_2023_36780_MOESM1_ESM.pdf]

Supplementary Figure 1

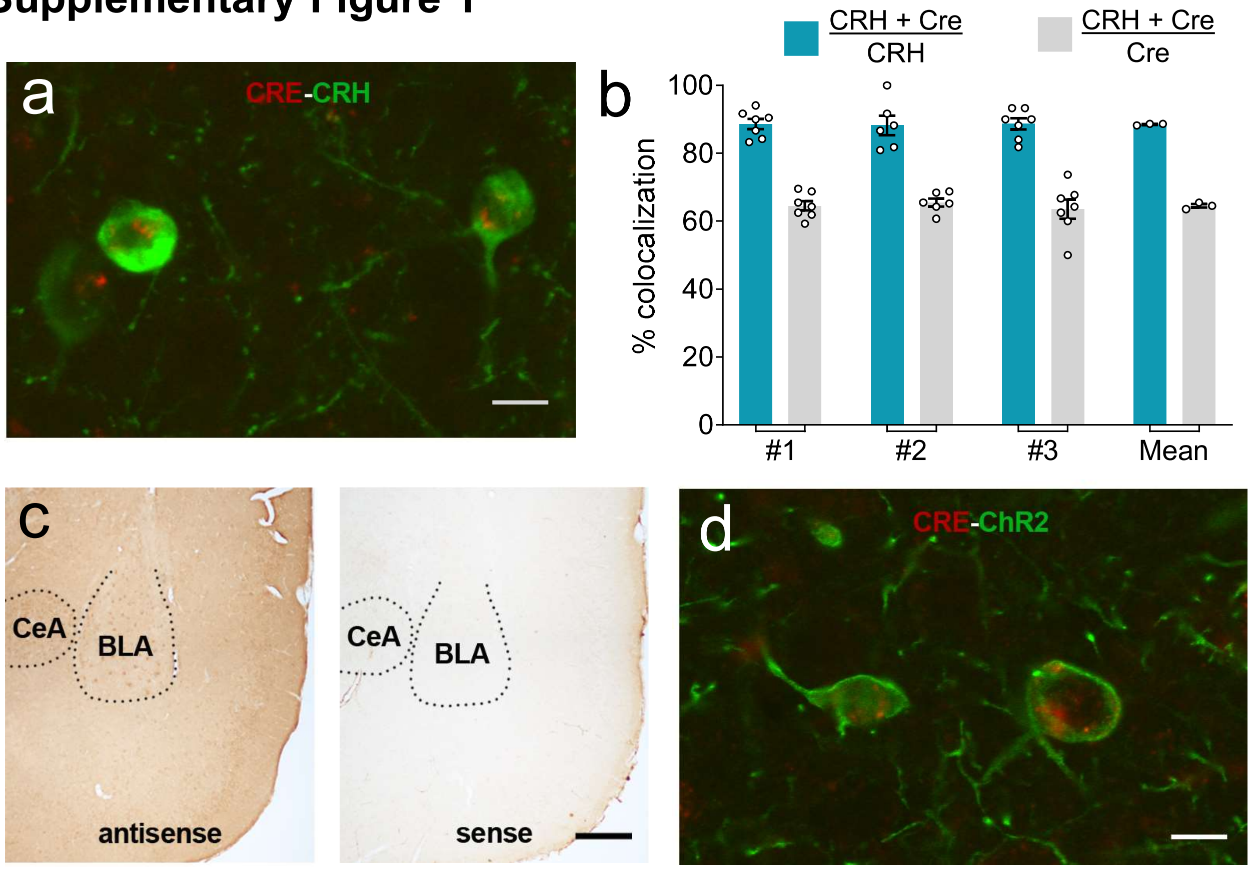

Supplementary Figure 1: Colocalized Cre expression in BLA neurons expressing endogenous CRH in the CRH-ires-Cre mouse line. (**a**) Co-expression of Cre in CRH neurons in the BLA is apparent, using combined *in situ* hybridization (Cre in red) and immunohistochemistry (CRH in green). (**b**) The colocalization of CRH and Cre, relative to total CRH in BLA is 88.51% ± 0.1453 (individual mice: #1, 88.64 ± 1.513; #2, 88.23 ± 2.869; #3, 88.67 ± 1.661). The colocalization of CRH and Cre, relative to total Cre is 64.52% ± 0.5572 (individual mice: #1, 64.54 ± 1.406; #2, 65.49 ± 1.205; #3, 63.55 ± 2.794). (**c**) Cre expression was detected in the BLA using a digoxigenin-5'-conjugated Cre RNA oligonucleotide probe. Signal was detected over BLA cell populations in sections exposed to the antisense probe, but not in those exposed to the sense probe. (**d**) Colocalized Cre expression (red) in ChR2-expressing neurons (green). The representative section is derived from a CRH-ires-Cre mouse receiving AAV1-EF1a-DIO-hChR2(h134R)-EYFP virus into the BLA. To confirm localization of Cre expression with endogenous CRH, virus injections and in situ hybridization were assessed in mice from two independent litters. Scale bar in **a** and **d** = 10 µm, in **c** = 280 µm. In **b**, bars represent mean ± SEM. #'s represent individual mice, dots represent analyzed brain sections. Teal bars represent % colocalization to total CRH, grey bars represent % colocalization to total Cre. Source data are provided as a Source Data file.

Supplementary Figure 2

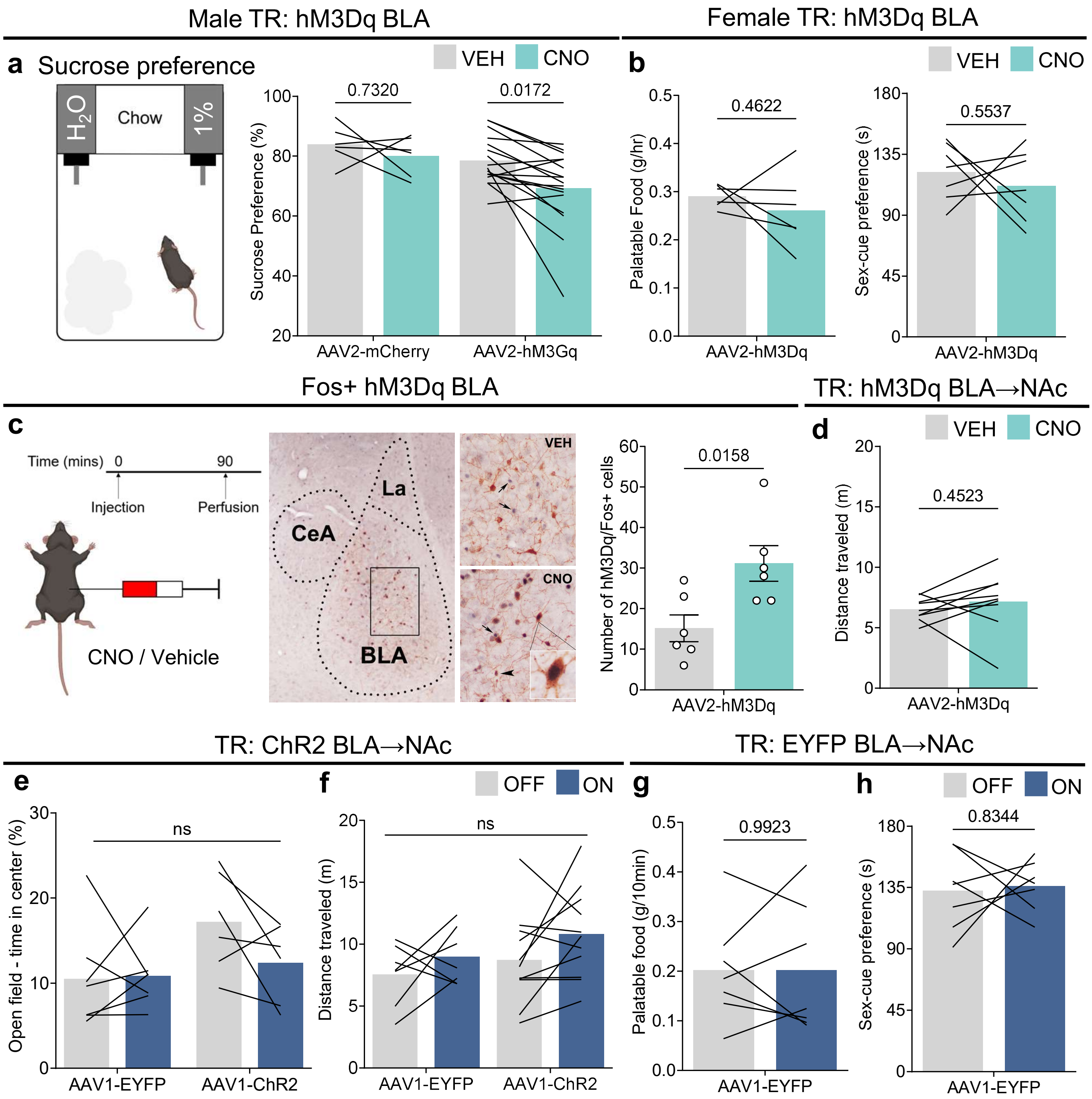

Supplementary Figure 2: Stimulating the CRH/GABA BLA→NAc projection suppresses reward. **a**, stimulating hM3Dq$^+$ CRH/GABA cells in the BLA decreased sucrose preference with systemic injection in males (*n* = 23 mice; *mCherry* = 7, *hM3Dq* = 16), **b**, but not females (hM3Dq - palatable food: *n* = 6 mice; sex-cue: *n* = 7 mice). **c**, Experimental timeline, example Fos expression in hM3Dq$^+$ CRH/GABA BLA neurons 90 minutes post injection of vehicle or CNO shows increased hM3Dq/Fos+ reactivity with CNO treatment compared to vehicle (*n* = 12 mice; *vehicle* = 6, *CNO* = 6). **d**, Microinjections of CNO in the medial NAc shell of mice to stimulate the hM3Dq+ CRH$^+$ BLA-origin projection did not affect distance traveled during the sex-cue task (*n* = 9 mice). **e, f**, Optical stimulation of the CRH$^+$ BLA→NAc projection did not alter **e**, time spent in center in an open field task (*n* = 13 mice; *EYFP* = 7, *ChR2* = 6) or **f**, distance travelled during the sex-cue task (*n* = 18 mice; *EYFP* = 7, *ChR2* = 11). **g**, **h**, Optical stimulation alone did not affect **g**, consumption of palatable food (*n* = 7 mice), or **h**, sex-cue preference (*n* =7 mice). In **a**, **b, d**-**h**, bars represent mean. Two-sided paired *t*-tests (**b**, **d**, **g**, **h**), two-sided unpaired *t*-test (**c**), two-way ANOVA with repeated measures followed by Sidak's post hoc test (**a**), two-way ANOVA with repeated measures (**e**, **f**). **a**, hM3Dq BLA: Virus - *F* = 6.075, DFn = 1, DFd = 42, *P* = 0.0179; post hoc with Sidak's multiple comparison (mCherry – *P* = 0.7320; hM4Di – *P* = 0.0172). **b**, hM3Dq BLA→NAc: Palatable food - *P* = 0.4622; Sex-cue - *P* = 0.5537. **c**, hM3Dq BLA Fos: *P* = 0.0158. **d**, hM3Dq BLA→NAc: *P* = 0.4523; **e**, ChR2 BLA→NAc: Light -*F* = 0.1873, DFn = 1, DFd = 23, *P* = 0.6692; **f**, ChR2 BLA→NAc: Light - *F* = 2.426, DFn = 1, DFd = 32, *P* = 0.1292; **g**, EYFP BLA→NAc: *P* = 0.9923; **h**, EYFP BLA→NAc: *P* = 0.8344. Grey = vehicle / light off, teal = CNO, blue = light on. Source data are provided as a Source Data file.

Supplementary Figure 3

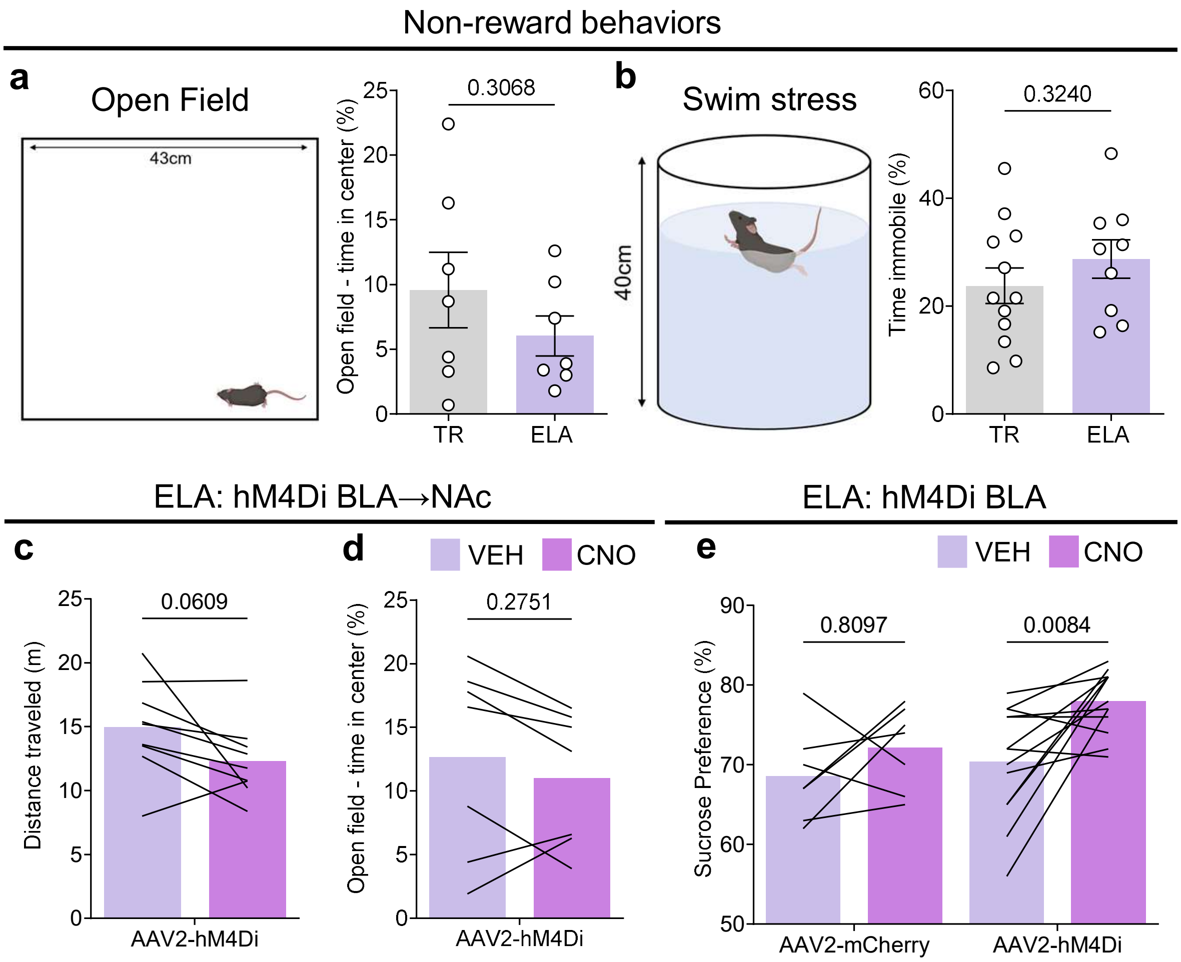

Supplementary Figure 3: Inhibiting the CRH/GABA BLA→NAc projection rescues reward deficits following early life adversity. a, b, ELA mice do not have altered response to stress-mediating, non-reward tasks, for example **a**, time spent in the center in an open field (*n* = 14 mice; *TR* = 7, *ELA* = 7) or **b**, immobility time during swim stress (*n* = 21 mice; *TR* = 12, *ELA* = 9). **c**, Inhibiting the CRH/GABA BLA→NAc projection did not affect distance traveled during the sex-cue task (*n* = 9 mice) or **d**, time spent in the center in an open field task (*n* = 7 mice). **e**, Inhibiting hM4Di$^+$ CRH/GABA BLA neurons enhances preference for sucrose in ELA mice (*n* = 20 mice; *mCherry* = 7, *hM4Di* = 13). In **a** and **b**, bars represent mean ± SEM. In **c-e**, bars represent mean. Two-sided unpaired t-tests (**a**, **b**), two-sided paired t-tests (**c**, **d**), Two-way ANOVA with repeated measures followed by Sidak's post hoc test (**e**). **a**, TR vs ELA: $P = 0.3083$; **b**, TR vs ELA: $P = 0.3240$; **c**, BLA→NAc hM4Di: $P = 0.0609$; **d**, BLA→NAc hM4Di: P=0.2751; **e**, ELA hM4Di BLA: Virus - $F = 4.25$, DFn = 1, DFd = 36, $P = 0.0465$; Drug - $F = 9.041$, DFn = 1, DFd = 36, $P = 0.0048$; post hoc with Sidak's multiple comparison (mCherry – $P = 0.8097$; hM4Di – $P = 0.0084$). Grey = typical reared, mauve = ELA / ELA + vehicle, pink = ELA + CNO. Source data are provided as a Source Data file.

Supplementary Figure 4

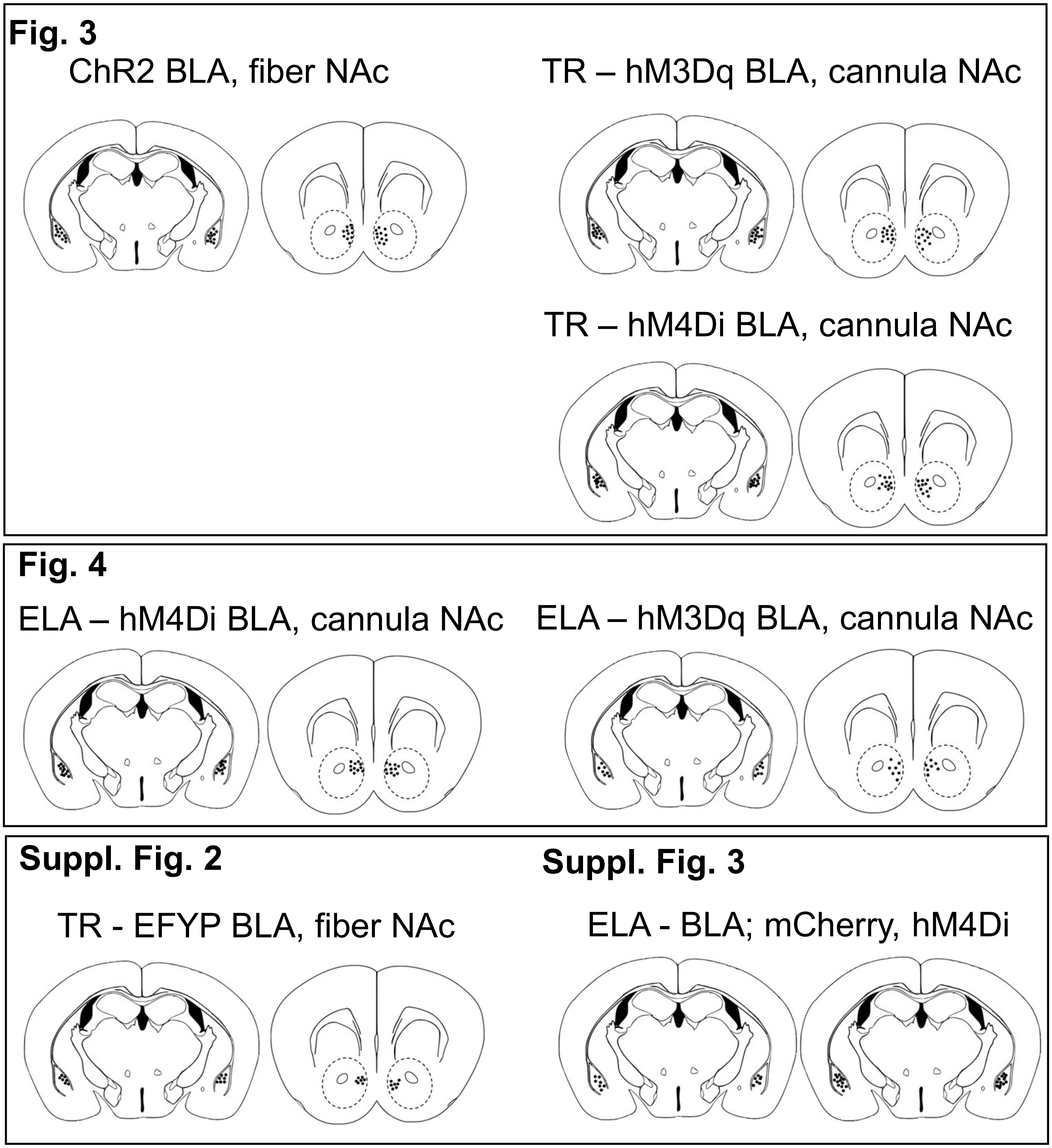

Supplementary Figure 4: Viral injection and cannula/fiber locations in the BLA and NAc. Schematic representations of bilateral viral injection sites and cannula/fiber implant terminal location positions.
