# Supplementary material for: Stress-induced plasticity of a CRH/GABA projection disrupts reward behaviors in mice
Source: Nat Commun. 2023 Feb 25;14:1088. doi: 10.1038/s41467-023-36780-x (PMC9968307; doi:10.1038/s41467-023-36780-x)
Supplement: Supplementary file 3 — Reporting Summary [file 41467_2023_36780_MOESM3_ESM.pdf]

nature portfolio

Corresponding author(s): Tallie Z. Baram, MD, PhD (tallie@uci.edu)

Last updated by author(s): Feb 7, 2023

Reporting Summary

Nature Portfolio wishes to improve the reproducibility of the work that we publish. This form provides structure for consistency and transparency in reporting. For further information on Nature Portfolio policies, see our Editorial Policies and the Editorial Policy Checklist.

Statistics

For all statistical analyses, confirm that the following items are present in the figure legend, table legend, main text, or Methods section.

n/a	Confirmed	
☐	☒	The exact sample size (*n*) for each experimental group/condition, given as a discrete number and unit of measurement
☐	☒	A statement on whether measurements were taken from distinct samples or whether the same sample was measured repeatedly
☐	☒	The statistical test(s) used AND whether they are one- or two-sided<br>*Only common tests should be described solely by name; describe more complex techniques in the Methods section.*
☐	☒	A description of all covariates tested
☐	☒	A description of any assumptions or corrections, such as tests of normality and adjustment for multiple comparisons
☐	☒	A full description of the statistical parameters including central tendency (e.g. means) or other basic estimates (e.g. regression coefficient) AND variation (e.g. standard deviation) or associated estimates of uncertainty (e.g. confidence intervals)
☐	☒	For null hypothesis testing, the test statistic (e.g. *F*, *t*, *r*) with confidence intervals, effect sizes, degrees of freedom and *P* value noted<br>*Give P values as exact values whenever suitable.*
☒	☐	For Bayesian analysis, information on the choice of priors and Markov chain Monte Carlo settings
☒	☐	For hierarchical and complex designs, identification of the appropriate level for tests and full reporting of outcomes
☒	☐	Estimates of effect sizes (e.g. Cohen's *d*, Pearson's *r*), indicating how they were calculated

Our web collection on statistics for biologists contains articles on many of the points above.

Software and code

Policy information about availability of computer code

Data collection	For behavioral analyses, Ethovision XT15 (Noldus, US) software was used for animal tracking. For electrophysiological recordings, data were collected using a Multiclamp 700B amplifier, Digidata 1550B (Molecular Devices, US) and Clampex 11 (Molecular Devices, US). For image acquisition, ImageJ2 and Photoshop CS5 (Adobe, US) were used.
Data analysis	For animal zone tracking, Ethovision XT15 (Noldus, US) software was used. For statistical analyses, GraphPad Prism (version 9) was used.

For manuscripts utilizing custom algorithms or software that are central to the research but not yet described in published literature, software must be made available to editors and reviewers. We strongly encourage code deposition in a community repository (e.g. GitHub). See the Nature Portfolio guidelines for submitting code & software for further information.

Data

Policy information about availability of data

All manuscripts must include a data availability statement. This statement should provide the following information, where applicable:

- Accession codes, unique identifiers, or web links for publicly available datasets
- A description of any restrictions on data availability
- For clinical datasets or third party data, please ensure that the statement adheres to our policy

All the raw and/or processed data presented in this manuscript are available upon request. All mouse illustrations included in the main and supplementary figures were created with BioRender.com. Source data are provided with this paper.

Human research participants

Policy information about studies involving human research participants and Sex and Gender in Research.

Reporting on sex and gender	Not applicable to this study
Population characteristics	Not applicable to this study
Recruitment	Not applicable to this study
Ethics oversight	Not applicable to this study

Note that full information on the approval of the study protocol must also be provided in the manuscript.

Field-specific reporting

Please select the one below that is the best fit for your research. If you are not sure, read the appropriate sections before making your selection.

☒ Life sciences ☐ Behavioural & social sciences ☐ Ecological, evolutionary & environmental sciences

For a reference copy of the document with all sections, see nature.com/documents/nr-reporting-summary-flat.pdf

Life sciences study design

All studies must disclose on these points even when the disclosure is negative.

Sample size	Sample size was determined based on previous studies from this lab and others to reliably reproduce the ELA phenotype. The sample size employed a minimum of 3 biological replicates, and from at least two different litters, to prevent possible litter effects. All sample sizes are listed in each figure legend.
Data exclusions	Mice were excluded from analyses only if virus location and/or guide cannula/optic fiber placement were not located in the basolateral amygdala and nucleus accumbens, respectively.
Replication	To confirm CRH+ BLA-NAc projection, virus injections and projection imaging was assessed in at least two different litters. In addition to this, all behavioral projection manipulations postmortem confirmed localization of virus in BLA and NAc.<br><br>To verify all behavioral data, experiments were replicated at least once and combined. Experimental animals were a combination from at least four different litters to ensure effects were not the result of one specific litter.
Randomization	On postnatal 2, mouse dams were randomly assigned to typical rearing or limited bedding and nesting cage environments. On postnatal day 60, the offspring were randomly assigned to virus groups. During behavior testing, mice were randomly assigned to vehicle/CNO treatment and light stimulation on/off order.
Blinding	All experimenters were blinded to animal groups throughout behavior experiments and analyses.<br><br>Experimenters could not be blind to maternal care scoring during postnatal days 2-9 when confirming early life adversity in the limited bedding and nesting environment as the environment is visibly different to the typical rearing cage environment.

Reporting for specific materials, systems and methods

We require information from authors about some types of materials, experimental systems and methods used in many studies. Here, indicate whether each material, system or method listed is relevant to your study. If you are not sure if a list item applies to your research, read the appropriate section before selecting a response.

Materials & experimental systems

n/a	Involved in the study	
☐	☒	Antibodies
☒	☐	Eukaryotic cell lines
☒	☐	Palaeontology and archaeology
☐	☒	Animals and other organisms
☒	☐	Clinical data
☒	☐	Dual use research of concern

Methods

n/a	Involved in the study	
☒	☐	ChIP-seq
☒	☐	Flow cytometry
☒	☐	MRI-based neuroimaging

Antibodies

Antibodies used

1. Anti-CRH antibody, courtesy of Paul E. Sawchenko (Salk Institute, PBL#rC68)
2. HRP anti-IgG antibody (cat # NEF812, Lot # 050841, PerkinElmer, US)
3. Anti-IgG_Alexa 488 antibody (cat # A-11034, Lot # 1971418, Invitrogen, US)
4. Anti-DIG antibody (Cat # MAB7520, Lot # CGBN0218061, R&D Systems, US)
5. Anti-IgG (biotinylated) (Cat # BA-9200, Lot # ZE0924, Vector Labs, US)
6. Anti-GFP antibody (Cat # 2555S, Lot # 2, Cell signaling, US)

Validation

All antibodies used in the study have been validated by the manufacturer, us, or published extensively by others. For example:

1. Anti-CRH antibody, courtesy of Paul E. Sawchenko (Salk Institute, PBL#rC68)

Chen Y, Molet J, Gunn BG, Ressler K, Baram TZ. Diversity of Reporter Expression Patterns in Transgenic Mouse Lines Targeting Corticotropin-Releasing Hormone-Expressing Neurons. Endocrinology. 2015 Dec;156(12):4769-80. doi: 10.1210/en.2015-1673. Epub 2015 Sep 24. PMID: 26402844; PMCID: PMC4655217.

Gunn BG, Sanchez GA, Lynch G, Baram TZ, Chen Y. Hyper-diversity of CRH interneurons in mouse hippocampus. Brain Struct Funct. 2019 Mar;224(2):583-598. doi: 10.1007/s00429-018-1793-z. Epub 2018 Nov 20. PMID: 30456559; PMCID: PMC6420853.

2. HRP anti-IgG antibody (cat # NEF812, Lot # 050841, PerkinElmer, US)
https://www.perkinelmer.com/uk/product/anti-rabbit-igg-hrp-labeled-goat-nef812001ea

Bouzaiene M, Angers A, Anctil M. Immunohistochemical localization of a retinoic acid-like receptor in nerve cells of two colonial anthozoans (Cnidaria). Tissue Cell. 2007 Apr;39(2):123-30. doi: 10.1016/j.tice.2007.02.001. Epub 2007 Mar 21. PMID: 17376496.

VVargas, V.E., Kaushal, K.M., Monau, T. et al. Long-Term Hypoxia Enhances Cortisol Biosynthesis in Near-Term Ovine Fetal Adrenal Cortical Cells. Reprod. Sci. 18, 277–285 (2011).

3. Anti-IgG_Alexa 488 antibody (cat # A-11034, Lot # 1971418, Invitrogen, US) Cited > 5500 times. Examples include:

https://www.thermofisher.com/antibody/product/A-11034.html?
gclid=Cj0KCQiA2-2eBhClARIsAGLQ2Rlgusubz2ZUf66nii1uAcepIgug6WzPH7nG9Ubb2c_QsKg9YruklBcaAhA2EALw_wcB&ef_id=Cj0KCQiA2-2eBhClARIsAGLQ2Rlgusubz2ZUf66nii1uAcepIgug6WzPH7nG9Ubb2c_QsKg9YruklBcaAhA2EALw_wcB:G:s&s_kwcid=AL!3652!3!516608152221!!!g!!!12825517856!122158235235&cid=bid_pca_aus_r01_co_cp1359_pjt0000_bid00000_0se_gaw_dy_pur_con

Sakai, W., Swisher, E., Karlan, B. et al. Secondary mutations as a mechanism of cisplatin resistance in BRCA2-mutated cancers. Nature 451, 1116–1120 (2008). https://doi.org/10.1038/nature06633

Sharma R, Al-Saleem FH, Panzer J, Lee J, Puligedda RD, Felicori LF, Kattala CD, Rattelle AJ, Ippolito G, Cox RH, Lynch DR, Dessain SK. Monoclonal antibodies from a patient with anti-NMDA receptor encephalitis. Ann Clin Transl Neurol. 2018 Jul 5;5(8):935-951. doi: 10.1002/acn3.592. PMID: 30128318; PMCID: PMC6093837.

4. Anti-DIG antibody (Cat # MAB7520, Lot # CGBN0218061, R&D Systems, US)

https://www.rndsystems.com/products/digoxigenin-antibody-611621_mab7520#product-details

5. Anti-IgG (biotinylated) (Cat # BA-9200, Lot # ZE0924, Vector Labs, US). Cited > 5500 times. Examples include:

https://vectorlabs.com/products/antibodies/biotinylated-goat-anti-mouse-igg

Lilly JL, Sheldon PR, Hoversten LJ, Romero G, Balasubramaniam V, Berron BJ. Interfacial polymerization for colorimetric labeling of protein expression in cells. PLoS One. 2014 Dec 23;9(12):e115630. doi: 10.1371/journal.pone.0115630. PMID: 25536421; PMCID: PMC4275217.

Hallett JM, Ferreira-Gonzalez S, Man TY, Kilpatrick AM, Esser H, Thirlwell K, Macmillan MT, Rodrigo-Torres D, Dwyer BJ, Gadd VL, Ashmore-Harris C, Lu WY, Thomson JP, Jansen MA, O'Duibhir E, Starkey Lewis PJ, Campana L, Aird RE, Bate TSR, Fraser AR, Campbell JDM, Oniscu GC, Hay DC, Callanan A, Forbes SJ. Human biliary epithelial cells from discarded donor livers rescue bile duct structure and function in a mouse model of biliary disease. Cell Stem Cell. 2022 Mar 3;29(3):355-371.e10. doi: 10.1016/j.stem.2022.02.006. PMID: 35245467; PMCID: PMC8900617.

6. Anti-GFP antibody (Cat # 2555S, Lot # 2, Cell signaling, US)

https://www.cellsignal.com/products/primary-antibodies/gfp-antibody/2555

Li Y, Fan T, Li X, Liu L, Mao F, Li Y, Miao Z, Zeng C, Song W, Pan J, Zhou S, Wang H, Wang Y, Sun ZS. Npas3 deficiency impairs cortical astrogenesis and induces autistic-like behaviors. Cell Rep. 2022 Aug 30;40(9):111289. doi: 10.1016/j.celrep.2022.111289. Erratum in: Cell Rep. 2022 Nov 8;41(6):111551. Erratum in: Cell Rep. 2022 Dec 6;41(10):111767. PMID: 36044858.

Ong YT, Andrade J, Armbruster M, Shi C, Castro M, Costa ASH, Sugino T, Eelen G, Zimmermann B, Wilhelm K, Lim J, Watanabe S, Guenther S, Schneider A, Zanconato F, Kaulich M, Pan D, Braun T, Gerhardt H, Efeyan A, Carmeliet P, Piccolo S, Grosso AR, Potente M. A YAP/TAZ-TEAD signalling module links endothelial nutrient acquisition to angiogenic growth. Nat Metab. 2022 Jun;4(6):672-682. doi: 10.1038/s42255-022-00584-y. Epub 2022 Jun 20. PMID: 35726026; PMCID: PMC9236904.

Animals and other research organisms

Policy information about studies involving animals; ARRIVE guidelines recommended for reporting animal research, and Sex and Gender in Research

Laboratory animals	Male and female B6(Cg)-Crhtm1(cre)Zjh/J mice. Common name - CRH-ires-CRE mice.<br><br>Early life adversity age: PN2-PN9<br>Viral injections/cannula/optic fiber placement: PN60<br>Projection quantification, electrophysiology, behavior: PN100-180
Wild animals	No wild animals were used in this study
Reporting on sex	Male and female mice were used in this study. In Figure 2, stimulation of the CRH+ BLA-NAc projection affected reward behaviors in males only. The rest of the study assessed males only due to this finding.
Field-collected samples	No field-collected samples were used in this study.
Ethics oversight	All experimental procedures were approved by the University of California-Irvine Institutional Animal Care and Use Committee (AUP18-183 and 21-128) and were in accordance with the guidelines from the National Institute of Health.

Note that full information on the approval of the study protocol must also be provided in the manuscript.
